# Complexity leadership in action: a team science case study

**DOI:** 10.3389/frma.2023.1211554

**Published:** 2023-07-27

**Authors:** Gemma Jiang, Diane Boghrat, Jenny Grabmeier, Jennifer E. Cross

**Affiliations:** ^1^Institute for Research in the Social Sciences, Colorado State University, Fort Collins, CO, United States; ^2^Imageomics Institute, The Ohio State University, Columbus, OH, United States; ^3^Translational Data Analytics Institute, The Ohio State University, Columbus, OH, United States

**Keywords:** complexity leadership theory, emergence, adaptive process, team science, team capacity development, effective meetings, enable cross-disciplinary research, teaming professionals

## Abstract

**Introduction:**

This team science case study explores one cross-disciplinary science institute's change process for redesigning a weekly research coordination meeting. The narrative arc follows four stages of the adaptive process in complex adaptive systems: disequilibrium, amplification, emergence, and new order.

**Methods:**

This case study takes an interpretative, participatory approach, where the objective is to understand the phenomena within the social context and deepen understanding of how the process unfolds over time and in context. Multiple data sources were collected and analyzed.

**Results:**

A new adaptive order for the weekly research coordination meeting was established. The mechanism for the success of the change initiative was best explained by complexity leadership theory.

**Discussion:**

Implications for team science practice include generating momentum for change, re-examining power dynamics, defining critical teaming professional roles, building multiple pathways towards team capacity development, and holding adaptive spaces. Promising areas for further exploration are also presented.

## 1. Introduction

Large science institutes face unique leadership challenges and opportunities. In this case study, we refer to the type of cross-disciplinary science institutes, in some instances referred as centers, that are formed upon the receipt of direct federal grant support. The initial funding period typically lasts 5 years, in some cases with an opportunity for renewal. They typically involve between 40 and 60 researchers from more than 10 U.S. higher education institutions and strive to meet the expectation of deep integration among diverse disciplines around a shared research mission that is of social and scientific importance.

The main challenges facing such institutes include a large, highly diverse membership charged with deep knowledge integration; unaligned goals among team members; permeable boundaries; geographic dispersion; and high task interdependence (National Research Council, 2015). These same challenges also present unprecedented opportunities to depart from the “business as usual” way of conducting science and instead develop new leadership culture, structures, processes, and practices that support and enable collaborative science. Among researchers from disparate disciplinary and institutional backgrounds, it is unlikely that one pre-existing way of doing things will presume to take precedence over others. To work together successfully, researchers within institutes are compelled to examine their assumptions and remain open to changes.

Complexity leadership theory (CLT) provides a theoretical framework for organizational leaders and members to lead change by enabling the adaptive process that drives new knowledge production, innovation, and adaptability (Uhl-Bien et al., 2007; Uhl-Bien and Arena, 2017; Uhl-Bien, 2021). Complex adaptive systems (CAS), a basic unit of analysis in complexity science, are neural-like networks of agents who interact within boundaries and form complex dynamics. CLT builds on assumptions of CAS that interactions among interdependent agents lead to nonlinear change, the results of which cannot be predicted. This phenomenon, called emergence—the appearance of new traits, properties, and patterns—explains the inherent potential for creativity in CAS (Cilliers, 2002; Johnson, 2012; Lichtenstein, 2014).

CLT outlines three types of leadership:

- **Entrepreneurial leadership** refers to the adaptive, creative, and learning actions that emerge from the interactive dynamics among individuals. It is a function of the entrepreneurial system responsible for experimentation, innovation, and novelty.
- **Operational leadership** refers to the actions of individuals and groups in formal authority roles who plan and coordinate activities to accomplish pre-determined goals and outcomes. It is a function of the operational system responsible for standardization, productivity and results.
- **Enabling leadership** focuses on the interaction between operational and entrepreneurial leadership and facilitates the adaptive process. Effective enabling leadership helps initiate and amplify support for novelty, innovation, change, and the eventual incorporation of novelty into the operational system to establish a new adaptive order.

In a system faced with adaptive pressure to change, these three types of leadership work together to pivot the system away from an order response (i.e., a state with no change) toward an adaptive response (i.e., a state with desired change). In the absence of enabling leadership, new ideas developed within the entrepreneurial system can easily hit the metaphorical brick wall and fail to integrate into the operational system.

This paper shares a case study in which a newly formed science institute's struggles with effective cross-disciplinary, integrative science meetings tipped the system toward disequilibrium. We describe a process of leading change and ultimately establishing a new adaptive order with redesigned meeting structure and processes. The narrative arc follows the four stages of the adaptive process as integrated by Uhl-Bien (2021) from CLT (Uhl-Bien et al., 2007) and generative emergence frameworks (Lichtenstein, 2014), and illustrates the three leadership types' involvement in each stage. We weave together the threads of science and leadership and highlight the importance of integrating these two distinct aspects that, like two wings of the same bird, are both necessary for any large collaborative science initiative to succeed.

## 2. Methods

This case study takes an interpretative, participatory approach, where the objective is to understand the phenomena within the social context and deepen understanding of how the process unfolds over time and in context (Yin, 2003; Elliott and Timulak, 2005; Guba and Lincoln, 2005; Crowe et al., 2011). The co-authors co-constructed the inquiry through examination of institute documents (meeting minutes describing the purpose and process of weekly institute meetings, monthly newsletters, team communication emails), observational notes from leadership meetings, informal interviews with participants, and personal reflection notes from the first author's reflective journal entries pertaining to team coaching and management. Additionally, we collected post-meeting assessment surveys which were implemented to gather participant feedback, guide the change process, and inform meeting improvement. Data examined in this case study include materials produced between August 2022 and April 2023.

The various data sources were reviewed and analyzed by three co-authors, in an iterative fashion where the data are looked at critically and reflexively for categories and themes, comparing interpretation across co-authors, and engaging in credibility checks at each stage (Elliott and Timulak, 2005). The focus of the analysis was to explore how team documents, reflections, and notes reflect or illustrate the concepts outlined by complexity leadership theory and also identifying the particularities of this case and how the process unfolded over time. On this illustrative case study, the focus of analysis was on understanding this case from multiple perspectives and illustrating how the tensions of entrepreneurial and operational leadership unfolded and how enabling leadership helped to manage the tension within this particular setting and change process.

## 3. The change process

An integrated framework from CLT (Uhl-Bien et al., 2007) and generative emergence (Uhl-Bien, 2021) describes the adaptive process in four stages, starting with disequilibrium caused by an adaptive challenge and ending with a new adaptive order that resolves the challenge. These four stages are: disequilibrium, amplification, emergence, and new order. Below, we describe the characteristics of each stage as operationalized in the change process in this case study.

### 3.1. Disequilibrium: pressures and tension

The adaptive process begins with disequilibrium when a system feels pressure to change. The pressures can come from external sources such as requirements from funders, or internal sources such as member dissatisfaction. These pressures are often classified as adaptive challenges that require new ways of thinking and behaving (Uhl-Bien, 2021).

During the summer of this institute's first year of funding, a new program director (second author) joined the team and was given responsibility for the group's weekly research coordination meetings. The institute leaders had initiated this meeting cadence when the institute was created, but there existed no stated purpose, objectives or strategies for implementing the meetings. Noting the challenge of low meeting engagement and ownership, she consulted with the team scientist (first author) and, with the institute leadership team's support, called for a town hall-style meeting to crowdsource ideas for a redesign.

### 3.2. Amplification: entrepreneurial and operational systems

In response to adaptive challenges, entrepreneurial leadership typically initiates an ideation process characterized by adaptive tension and task-related conflicts, in which innovative team members identify and experiment with different pathways, and their ideas conflict, combine, and recombine until potential adaptive responses are identified. The opposite of adaptive responses are order responses that fail to take advantage of the adaptive challenge. This can happen by an operational system introducing quick fixes to reduce the discomfort that comes with disequilibrium or avoiding it altogether and simply wishing it away. Actors in CAS have choices in how they respond to an adaptive challenge, ranging from adaptive responses to order responses, but these choices are not always made consciously or intentionally (Uhl-Bien, 2021). Actors in operational systems that have access to resources and decision-making authority can play an important role in bringing about adaptive responses, as will be discussed in section 3.3.

Following the town hall, the team scientist assumed responsibility for leading the change effort to redesign the meeting series, and in doing so exercised primarily entrepreneurial leadership.

The team scientist collected and synthesized data from the sense-making process, which included the town hall, follow-up conversations, and email exchanges; these are presented in detail in section 3.2.1. Drawing on her facilitation expertise, she devised a new design that addressed most of the needs articulated and integrated most of the ideas expressed. She presented the design to a subgroup of senior institute leaders representing operational leadership, where she received comments such as “this design is too complicated” and “do not overthink it.” She recognized these comments as order responses that, if actualized, would not fully capitalize on the opportunity presented by this adaptive challenge and the creative ideas of institute members who participated in the redesign process.

#### 3.2.1. Summary of collective sense-making data

Feedback from institute members during the sense-making process reflected several themes centered on “what to talk about.” Participants detailed a need for **fostering collaboration** by “identify[ing] connection points [and] possible areas of overlap” and **developing institute coherence**, which one member described as “things that make us an institute instead of a collection of projects.” Members indicated a desire for **project coordination** to “learn about the latest cool thing from a project I am not involved in” and **knowledge integration** across projects, such as “talk[ing] about research from different perspectives so that everyone can stretch beyond their comfort zone” and learning about relevant research outside the institute. Needs were expressed for **capacity building** through tutorials that “members of different groups can choose to attend and be acquainted with the core basics of different fields, and they would know whom to contact for help.” Lastly, members expressed a desire for **collaborative problem solving** by “learn[ing] about the challenges researchers are having that I could help with” and “discussing issues of concern to the entire community.”

Important insights were also shared regarding “how to organize this.” Members voiced interest in a more member-directed approach, with an emphasis on **rotating topics/projects** by “dedicat[ing] specific weeks to specific topics” and “on a rotating basis, giv[ing] (responsibility for planning) to individuals and let[ting] them decide what to do.” Participants also expressed needs for **more conversation** instead of reporting, with one member recommending that the group “focus meetings on conversations, not on updates.” Another person stated a need for **a multi-week agenda** to plan ahead.

Institute members also expressed dissatisfaction with how meetings were originally conducted, which yielded important insights about what not to do. Their dissatisfaction included: using a top-down structure, spending too much time on bureaucracy, having an unclear decision-making process, and not allowing talking space for students and postdocs.

#### 3.2.2. Task conflicts and emotional labor

The initial responses from senior institute leaders, such as “this design is too complicated,” are a type of order response to change that is best characterized as a task-related conflict with the entrepreneurial leaders. In this context, the conflict signaled two divergent visions about the future of the meeting series. According to complexity leadership theory, task-related conflicts can be beneficial because they compel groups to engage heterogeneous perspectives, and new ideas are born through the process of conflict and connection (Uhl-Bien, 2021).

However, there is an emotional aspect of the story, too, which can make the difference between falling back into an order response or leaping forward into an adaptive response. Even with a clear conceptual understanding of task-related conflicts and resistance, the emotional discomfort is still difficult to manage. The team scientist's journal recorded the incident in an entry entitled “Somebody has to be in pain to make this happen. Why not me?” The pain was exacerbated by the uneven power dynamics related to age, gender, race, disciplines, and roles within the institute, which will be discussed further in section 4.2.

Buoyed by the enthusiastic participation of institute members and the many excellent ideas shared in the sense-making process, the entrepreneurial leaders' determination to carry this through prevailed over discomfort, and the story continued. A question to reflect on could be: How many change initiatives fail because of the emotional costs involved?

### 3.3. Emergence: enabling leadership

Enabling leadership creates the conditions for integrating an adaptive solution into the operational system. In this stage, enabling and entrepreneurial leadership work together to refine key ideas until they can be adopted by the operational system and more broadly applied. This continuous adaptation is an expected part of the emergence process. If adaptive solutions fail to integrate with the operational system, they remain local to a small group of innovators and are eventually forgotten (Uhl-Bien, 2021).

After meeting with the senior institute leaders, the team scientist transitioned her primary leadership function from entrepreneurial to enabling and turned her focus to integrating the innovative ideas of other entrepreneurial leaders. She engaged institute members who were vocal about their support for the redesign initiative and willing to exercise entrepreneurial leadership, and through one-on-one and small group consultations further refined the design into an adaptive solution that addressed several key challenges: engaging the next generation of scientists to increase participation, bringing in external speakers to build a broad intellectual base, creating space for collective problem solving, and leaving room for new ideas to surface. Key design features included: a monthly cycle with a featured research theme and weekly foci; formation of production teams with clearly delineated roles; and a monthly newsletter preview of an entire month's agenda. Additional details about the redesign can be found in the blog post *An effective way to organize research coordination meetings* (Jiang et al., 2023).

### 3.4. Stabilizing feedback: new order

Stabilizing feedback is the last stage in the adaptive process. Entrepreneurial and/or enabling leaders link with operational leaders to incorporate the adaptive solution into the operational system, and it operates in the form of a new adaptive order, with new processes, procedures, and/or products (Uhl-Bien, 2021).

The team scientist presented the adaptive solution to the full leadership team responsible for making institute-wide decisions. She framed the meeting redesign as a platform for institute coherence and cohesion as well as an opportunity to exemplify collaborative culture, and after fielding questions, noting further feedback, and assuring continuous improvement, she invited the leadership to vote on the redesign using the gradient of agreement scale (Kaner, 2014). The adaptive solution received overwhelming support and was adopted. In this process, the institute's leadership team exercised operational leadership by endorsing the adaptive solution, and the team scientist exercised enabling leadership to see to its integration into the operational system.

Since then, the weekly meeting's new design has gone through five monthly cycles, with one production team for each month and 20 weekly research coordination meetings in total.

#### 3.4.1. The change: before and after

The dramatic changes from before the meeting redesign and after are captured in five aspects: meeting preparation, meeting facilitation, participant engagement, attendance numbers, and the learning loop for meeting organizers (see Table 1).

**Table 1:** The change: before and after.

	**Before/The initial order**	**After/The new order**
Meeting preparation	A sign-up sheet rarely utilized, no invited speakers, no information on agenda before meeting, solicitations for content went unanswered	Production teams were assembled that aligned with specific themes to prepare content; invited speakers were confirmed before newsletter came out; newsletter shared four weeks of agendas in advance
Meeting facilitation	Unclear who was facilitator, no set plans due to lack of input from institute members	Facilitator was designated and facilitation plans were prepared in consultation with presenters and speakers
Participant engagement	Most participants had their cameras turned off, and chats were quiet; little interaction with speakers/presenters or among participants	Most participants had their cameras turned on and were active during facilitated interactive time, chats were busy
Number of participants	Approximately 10 on average	Approximately 25 on average
Learning loop for meeting organizers	No learning loop	Participants' feedback was captured via post-meeting assessment surveys and informal conversations and integrated into content and facilitation for coming meetings

#### 3.4.2. Post-meeting assessment data

A short, anonymous survey to gather immediate feedback from meeting participants was administered after select meetings representing different meeting types and research themes. To avoid overburdening respondents, data were collected on eight meetings out of 20. In total, 28 responses were received.

The first and second questions were rated on a Likert scale of 1–5. The average score for the first question, “How helpful do you find the content of the meeting (i.e., what we talked about),” was 4.38. The average score for the second question, “How helpful do you find the facilitation of the meeting (i.e., how we spent time together),” was 4.34. These responses indicated that participants were generally content with the meeting redesign.

The third and final question was open-ended, with the prompt “Please kindly share any compliment, suggestions, or questions you might have.” The compliments submitted indicated the new design met most of the needs expressed in the sense-making process. One person remarked, “I really like the engagement. I wish we had more time to talk.” Another person shared, “It was nice to hear about projects I am not personally involved in, and I think it's good having the breakout rooms for people to get a deeper dive on the related topics they are interested in.”

Suggestions that informed timely adjustments were also received. One participant, for example, pointed out the ineffectiveness of breakout rooms in a session featuring an invited speaker: “The breakout groups have not been useful to me when there is an external speaker. I would rather have a general Q and A or discussion time right after the talk with everyone in the same room.” Another participant's comments suggested the inverse for sessions discussing research projects: “I thought the meeting was very well done. I would recommend more time in the breakout rooms. The sharing at the end seems rushed. I wonder if just giving that time to the breakout rooms would have been better and refer everyone to notes about each breakout room if they are curious.” Incorporating these comments led to more effective deployment of facilitation processes and better meeting experiences for all.

### 3.5. Case study summary

This case study operationalizes collaborative leadership by connecting a successful change initiative with complexity leadership theory, which helps to explain the mechanism of how the new adaptive order was established.

Unlike centralized decision making in most bureaucratic organizations, collaborative leadership calls for distributed sense making, decision making, and action taking (Jiang, 2023). The case presented here operationalized this principle of collaborative leadership by involving different stakeholder groups, employing different strategies, and supporting different types of leadership behaviors at different stages of change. This distributed and differentiated way of moving through the change process avoided the common pitfalls of an order response to adaptive pressure. Table 2 below summarizes this change process.

**Table 2:** Summary of the change process.

	**Purpose**	**Strategies**	**Stakeholders involved**	**Types of leadership**	**Change process**
Sense making	To understand needs and gather design ideas	Institute town hall, small group conversations, email correspondence	All institute members	Entrepreneurial and enabling	Disequilibrium amplification, and emergence
Decision making	To make decisions on the new meeting design	Presentation, consultation, voting	Institute leadership team	Operational and enabling	Tipping point toward new order
Action taking	To make continued adaptations and improvements	Production team consultations, meeting facilitation, monthly newsletter announcements	Weekly production team; all institute members	Entrepreneurial and enabling	New order

## 4. Implications for practice

This case study presents important implications for team science practice that point to promising possibilities for the future of science institutes when researchers and teaming professionals co-create leadership solutions.

### 4.1. Momentum for change

Establishing a sense of urgency is a necessary condition for successful change initiatives (Kotter, 2012). In the absence of change momentum, change efforts are more likely to run into resistance and never progress beyond the disequilibrium stage. Therefore, it is important for change leaders to leverage and amplify existing momentum toward change.

Momentum for change in this case was generated by a shared discontent with how the weekly meetings were operating and amplified by the new program director who was committed to recognizing and naming the disequilibrium and making improvements. At the time of this writing, the authors are again re-envisioning the meeting series design as an outcome of the institute's second annual all-hands meeting; at that gathering several significant needs surfaced that spurred momentum for a new change initiative around the research coordination meetings to accommodate the institute's continuing organizational development and evolving priorities.

It is important to note that the main takeaway for this case study is not one particular way of conducting research coordination meetings. Rather, it is to recount the successful application of a change theory with impactful outcomes. Any adaptive solution must be responsive to the situational contexts in which it exists. Even with seemingly similar challenges related to meeting design, a different team will apply the same theory and come up with a different meeting model. And for the same team, adaptive solutions for effective research coordination meetings will look different at different developmental stages. It is important to be mindful of the nonlinear nature of CAS and be adaptive in addressing emergent challenges.

### 4.2. Power dynamics

While success for large science teams hinges on collaborative leadership, most National Science Foundation-funded science projects default to principal investigators owning final decision-making authority. This practice creates a challenging, even threatening power dynamic for other team members to navigate when leading a change initiative. The questions, then, become: How can decision-making authority be distributed? Must it always be dictated by a hierarchical structure that presumes funded scientists have more authority than professional staff, and senior scientists have more power than junior ones?

We recommend an increase in concerted efforts around leadership development for senior scientists to enable their transition from individual achievers to effective, productive leaders–a sentiment echoed by the National Research Council's (2015) for translating and extending the leadership literature to create development opportunities for science leaders. Further, the historic particulars of academic research and science in general require that such leadership development efforts include in their subject matter identifying and mitigating social and organizational power dynamics.

Broadening participation in science is an ongoing issue, as problems with retention of women and other underrepresented groups continue to persist (National Center for Science and Engineering Statistics, 2021). Social power dynamics help perpetuate this problem, evidenced by reports of microaggressions experienced by students, staff, and faculty based upon their marginalized social identities related to race, sex, age, educational attainment, organizational role, and sexual orientation (Grossman and Porche, 2013; Young and Myron, 2015; O'Meara et al., 2017). Social identities impact whether an individual is viewed as a leader (Hogg, 2001) with authority to effect change within an organization.

Power dynamics similarly impact the ability of professional staff to drive change within higher education organizations. Traditional academic hierarchical structures place staff at lower levels (Rosser, 2004; Young and Myron, 2015; Bowles, 2022), which leads to a failure to consult them in change-making processes (Rosser, 2004). This is contraindicated given the critical roles of professional staff in identifying the need for change and executing implementation plans. We call for increased and codified recognition of the leadership roles of teaming professionals such as program directors, team scientists, and research strategists, and greater degrees of situational decision-making authority for these roles in efforts that are necessary for successful team science. Their often diverse disciplinary backgrounds, including social sciences and leadership studies, bring divergent perspectives to the exploration of new ways of conducting team science that are very much needed in the often homogenous science populations (Van der Vegt and Janssen, 2003; Hong and Page, 2004; National Center for Science and Engineering Statistics, 2021).

The success in this case study was co-created by the professional teaming staff and principal investigators of the institute. After some initial resistance, the principal investigators were willing to try the new meeting design, and they strengthened the new approach with their suggestions. Two variables that may have enabled this shifting of power dynamics to afford room for teaming professionals to assume leadership roles in the change initiative were: the aspiration among principal investigators to create a non-hierarchical leadership structure within the institute; and previous successful collaborations between the scientists and teaming professionals that engendered trust in the latter's abilities. The follow-on redesign process mentioned in 4.1 indicates a further deepening of trust, with the adaptive process proceeding more smoothly and without pushback as a result.

### 4.3. Defining critical roles

*The Fundamental Characteristics of a Translational Scientist* (Gilliland et al., 2019) named seven characteristics necessary for success as a translational scientist. Though these characteristics are developed based on studies of researchers in the medical field, they can be applied to articulate roles necessary for successful convergence endeavors in other research domains. Of these roles, some are best filled by researchers trained in specific research domains, such as domain expert and rigorous researcher. Other roles, such as process innovator or skilled communicator, call for critical examination of new functions and types of expertise needed on large science teams. These roles often require in-depth training and experience in social science subjects such as leadership, management, organizational psychology, communication, and sociology. Connecting with 4.2, such process innovator and skilled communicator roles are critical both for leading and supporting functions of a cross-disciplinary science team.

This case study highlights in particular the role of a process innovator, which provides process expertise and maintains sight of the operational objective for the scientists who are deeply immersed in their own disciplines. For this specific change initiative, the team scientist adopted the “chief doing officer” title coined by Strategic Doing (Morrison et al., 2019), a school of thought for agile leadership. As such, she led the group through the collective sense-making and decision-making processes, and during the action-taking stage served as the connective tissue among different scientists and science teams and nudged them for deliverables. The process innovator role is both a leadership and supporting role.

Large science teams are advised to explore other innovative roles as well, such as SCRUM Master (Sutherland and Sutherland, 2014) from the agile movement, which can share the work of leading the collaborative process with scientists. This case study classifies these roles under the umbrella of “professional teaming staff” and emphasizes the important functions they perform in the operation of science institutes. Other team science scholars have articulated these roles with titles such as “interdisciplinary executive scientist,” “research development professional,” and “integration and implementation specialist” (Hendren and Ku, 2019). These roles encompass a set of leadership, operational, and research tasks focused on the boundary work at the interface of different research units. The authors' experience confirms the need to articulate these roles and to incorporate the nuances from actual lived experiences of such roles. Further practice of and experimentation with approaches to meeting the evolving needs of complex science collaborations will provide additional clarity.

### 4.4. Multiple pathways toward team capacity development

When it comes to building capacity for collaboration, most science teams lean heavily into workshops that focus on specific team competencies but are detached from the team's working contexts. In contrast, in this case study complexity leadership principles were operationalized in the adaptive processes within the real-world work context of the teams.

The workshop approach does check a box and looks good in an annual report to funders. Beyond that, however, it is unclear how effective it is in fostering new learning behaviors and effecting real change. Bersin (2008) suggests that the addition of informal learning activities post-workshop may improve outcomes, with as much as 70% of job-relevant learning occurring as on-the-job learning, 20% occurring prior to formal training programs, and 10% occurring during training.

Follow-on activities after major training events can employ a variety of modalities, such as team coaching (Wageman and Lowe, 2019), team facilitation (Kahane, 2021), and specific interventions that center the team's developmental needs such as collaboration planning (Hall et al., 2019). As illustrated in the case study, the key is to remain grounded in the social context of the teams.

The need for contextual grounding also points to some of the nuances within the critical teaming staff function outlined in section 4.3. In addition to mastery of their content areas and the ability to convey highly abstract concepts in workshop settings, these personnel need the ability and opportunity to dwell in the trenches with science teams and be present as needs emerge in order to facilitate on-the-job capacity building and help create enabling conditions for the desired outcomes. In other words, they must be substantively involved in the situational contexts of the science teams they serve. The most effective paths forward will come not from a silver bullet from the professional teaming staff, nor from maintaining the status quo of established science practices. Rather, they will be co-created by scientists and professional teaming staff with a deep respect for situational contexts.

### 4.5. Adaptive spaces

Of the three types of leadership outlined in complexity leadership theory, science teams typically demonstrate strong entrepreneurial leadership in developing new ideas and pushing the boundary of discovery. They also commonly give requisite attention to operational leadership. Traditional operational leadership structures typically include full- or part-time administrative staff such as program managers and coordinators, communication managers, a leadership team overseeing administration, and an external advisory committee. However, the enabling leadership that can connect entrepreneurial and operational leadership and systematically support the emergence of new ideas and establishment of new orders is left unaddressed. One avenue to operationalizing enabling leadership intentionally is to establish “adaptive spaces”—dynamic and fluid social spaces where emergent challenges are addressed with adaptive processes (Uhl-Bien, 2021).

The authors of this case study have found meetings to be among the most effective adaptive spaces. Like a music ensemble's rehearsal before a show, or a sports team's training for a game, meetings are social spaces where a science team practices dynamic interactions that can lead to emergent outcomes. How a meeting is conducted is a microcosm of how a project is conducted; thus, each meeting is a potentially high-impact, low-difficulty intervention point to increase team effectiveness.

Yet science teams often do not expend adequate effort in leading effective meetings. Some meetings overemphasize operational leadership and trend toward overcontrol with status updates and presentations; others overemphasize entrepreneurial leadership and trend toward under control, with multiple sub-tracks of conversations that lack clear relevance or value for everyone present (Lipmanowicz and McCandless, 2013). Typical behaviors that we have observed in ineffective meetings include: faculty dominating the conversation, with little room for students' or administrators' input; agendas dominated by sub-group progress updates with little time for interaction or collective problem solving among the groups; two or three individuals steering the group conversation off-track while the rest become disengaged and even angry; researchers presenting their own research without considering their audience's capacity or involving their participation.

Many practices contribute to evolving a dysfunctional team meeting into an adaptive space. Concerted effort in organizing, planning, facilitating, and reflecting on meetings, as described in this case study, is a good place to start.

## 5. Conclusions

This case study is a novel application of complexity leadership theory to guide the change process in a team science context. The cross-disciplinary science institute's new adaptive order for its weekly research coordination meetings was co-created by scientists and professional teaming staff in service of science integration. The possibilities created by working together and leveraging social science principles to advance cross-disciplinary research are promising.

Further investigation of the following areas is recommended to capitalize on the successful outcome of this case study:

- The application of the complexity leadership framework in diverse team science contexts;
- The complexities of professional teaming staff roles;
- Development of diverse pathways for team science capacity building;
- Discovery of enabling conditions for adaptive spaces to exist and yield positive outcomes;
- Experimental approaches for addressing emergent challenges.

## Data availability statement

The original contributions presented in the study are included in the article/supplementary material, further inquiries can be directed to the corresponding author.

## Author contributions

GJ and DB contributed to the conception and design of the institute meeting format. GJ wrote the first draft of the manuscript. GJ, DB, and JEC wrote sections of the manuscript. All authors contributed to manuscript editing and revision and approved the published version.
